# Recovery effect of self‐myofascial release treatment using different type of a foam rollers

**DOI:** 10.1038/s41598-024-66577-x

**Published:** 2024-07-09

**Authors:** Bartłomiej Michalak, Anna Kopiczko, Robert Gajda, Jakub Grzegorz Adamczyk

**Affiliations:** 1 Department of Theory of Sport, Józef Piłsudski University of Physical Education in Warsaw, Marymoncka 34 St., 00-968 Warsaw, Poland; 2 Department of Human Biology, Józef Piłsudski University of Physical Education in Warsaw, Marymoncka 34 St., 00-968 Warsaw, Poland; 3 Center for Sports Cardiology, Gajda-Med Medical Center in Pułtusk, 06-100 Pułtusk, Poland; 4 Department of Kinesiology and Health Prevention, Jan Dlugosz University, 42-200 Czestochowa, Poland

**Keywords:** Myofascial release, Regeneration, Lactate clearance, Creatine kinase, Physiology, Health care

## Abstract

Among athletes, foam rolling is popular technique of myofascial release aimed to support recovery processes and counteract delayed onset muscle soreness. However, there is no consensus on the optimal parameters of the roller texture used in the procedure. The study aimed to determine whether using rollers with different textures and hardness (smooth/soft, grooved/mid, serrated/hard) in myofascial release affects post-exertional restitution rate and the level of perceived DOMS (Delayed Onset Muscle Soreness) after intense anaerobic exercise. The study involved 60 healthy and physically active men randomly divided into three experimental groups and one control group (passive rest)—each consisting of 15 individuals: STH—rolling with a smooth roller; G—rolling with a grooved roller; TP—rolling with a serrated roller; Pass—passive rest group. After performing a exercise test (one-minute high-intensity squat), blood lactate (LA), creatine kinase (CK) and pain perception (VAS Scale) were monitored. The analysis of the average LA concentration in the blood 30 min post-exercise showed a statistical difference for all rolling groups compared to the passive rest group: STH (p < 0.001), G (p < 0.001), TP (p = 0.035). No statistically significant differences were found between the CK measurement results in individual assessments. Statistically significant differences in VAS values were observed between G (p = 0.013) and TP (p = 0.006) groups and the Pass group at 48 h, as well as between STH (p = 0.003); G (p = 0.001); TP (p < 0.001) groups and the Pass group at 72 h. Based on statistical data, a strong influence (η^2^ = 0.578) of time on the quadriceps VAS variable was noted. The research results confirm the effectiveness of rolling in supporting immediate and prolonged recovery. The conducted studies indicate a significantly better pace of post-exertional recovery after a rolling procedure lasting at least 120 s. The texture and hardness of the tool used did not matter with such a duration of the treatment.

## Introduction

Optimizing sports training, preventing negative effects of effort, and minimizing the risk of injuries are important aspects of the training process. Effective post-exertion recovery is a key condition for enhancing athletes' performance, serving as a significant factor supporting adaptation^1^. One increasingly popular form of post-exertion recovery support is the Muscle-fascial release technique, which has been described in the literature as a type of manual therapy in which muscular and other soft tissues undergo pressure. In the literature, this term is referred to by the acronym MFR (Myofascial Release)^2^. An extension of this method is technique known as SMFR or simply SMR (Self-Myofascial Release)^3^ which has been proven that even a single session can be useful in supporting training^4^.

In practice, there are many devices with varied structures and hardness available on the market, with the most popular being foam rollers (FR). Due to their shape and size, foam rollers allow for the application of appropriate pressure while covering a relatively large surface area that you want to treat^5^. Additionally, it is believed that the wide variety of FR textures (more pronounced with spikes/knobs) enables more precise and deeper impact^6^. Other tools used for self-massage include massage sticks, massage guns, foam rollers with vibrating mechanisms, and lacrosse balls. For smaller areas of the body, mini foam rollers, golf balls, or tennis balls can also be used^7^.

The wide variety of foam rollers can be confusing, making it challenging to determine which one is most effective for a specific purpose. Based on the literature review, a small number of studies comparing the effects of different types of rollers were identified. For instance, a smooth-surfaced roller (STH) was used by MacDonald, Healey or Shu^8–10^. In all of these studies, it was found that rolling alleviates pain sensations, reduces feelings of fatigue, and even leads to the reduction of muscle inflammation. Cheatham and Stull^11^ compared the immediate effects of SMR treatment using rollers with three different densities (Soft—soft, MED—medium density, Hard—hard). All tools used had a Grid—G type texture (with grooves and small protrusions). In their experiment, they obtained significantly better results in terms of knee joint mobility and the perceived pressure pain threshold (PPT) in the quadriceps muscle compared to measurements taken before the intervention with the roller. Adamczyk et al.^12^ compared the effects of rolling with Grid and Smooth textured rollers, both with medium-density foam material. According to researchers, muscle and fascial relaxation alone using a roller seems to be an effective method in increasing lactate removal and counteracting DOMS, but the effect of the type of foam roller does not seem to have been sufficiently studied.

Furthermore, in the literature, experiments comparing foam rollers with massage sticks (MS) can be found. The outer material of these tools is typically hard and made of plastic^13^. DeBruyne et al.^14^, based on the analysis of 4 experiments, did not show significant differences between the obtained effects on the flexibility of the hamstring muscles between FR and MS. Additionally, they found the equivalence of rolling treatments to the effects achieved through static stretching. Moreover, in the literature, there are studies comparing traditional foam rollers with rollers equipped with a vibrating mechanism. Romero-Moraleda et al.^15^ demonstrated a slightly higher reduction in pain sensations measured using the VAS (Visual Analog Scale) in devices that enhanced massage effects with programmed vibration compared to regular FR. On the other hand, Ruggieri et al.^16^ did not find differences between a regular and a vibrating roller.

Numerous studies can be found that assess the immediate effects of SMR treatment using a single type of roller on joint range of motion (ROM) and muscle flexibility. In this matter, there is consensus among scientists, whose findings confirm the beneficial impact of rolling on ROM and muscle flexibility despite the use of different types of rollers^17^. Furthermore, scientists' interest has also focused on evaluating changes in physical fitness after roller application. Regarding this issue, the conclusions are not uniform. There are studies where a positive effect of rolling on speed is observed^18^, no impact in fitness tests^19^, or isometric contraction strength and vertical jump^8^. Research has also noted instances where the SMR procedure negatively affected performance^20^.

Based on literature review although evidence seems to justify the widespread use of foam rolling, the practical application of this treatment encounters the problem of a lack of clear recommendations^21^. The modulating factor in the effectiveness of rolling appears to be the type of foam roller used for the treatment. Increasing the local pressure on the massaged tissue by using harder tools can affect the analgesic effect. A non-smooth structure (grid) may also contribute^22^. Acting with a structure other than a smooth one may also be associated with the release of trigger points, increasing pressure on the tissue may improve recovery^23^, however, this hypothesis must be verified. An analysis of the literature reveals a lack of consensus on the optimal parameters of roller texture for the effectiveness of the applied technique, as well as the limited number of studies comparing the effects of rolling with different rollers in a single experiment. To fill this gap the aim of the study was to examine whether the use of foam rollers with different textures and hardness (smooth/soft, grooved/mid, trigger point/hard) in myofascial release enhances the post-exertional recovery rate and the perception of delayed onset muscle soreness (DOMS) after intense anaerobic exercise.

## Material and methods

### Participants

The study enrolled 60 healthy and physically active men and all participants provided written informed consent to participate in the study. Study was performed in line with the principles of the Declaration of Helsinki. Approval was granted by the Józef Piłsudski Ethics Committee (decision no. SKE 01-41/2016). Participants were informed about the risks associated with high physical exertion, as well as the procedures and requirements related to participating in the test.

Individuals participating in the experiment were required to have no contraindications to performing strenuous physical efforts or undergoing SMR treatment with a roller, such as thrombosis, varicose veins, bruises, fresh made tattoos, etc.^24^. Additionally, individuals who had experienced musculoskeletal injuries within the last 6 months from the date of the study, completed the rehabilitation process, or had any other conditions limiting physical activity were not eligible for the experiment^25^. The study included only a group of men, due to the inability to control the phase of the menstrual cycle, which affects endocrine activity, recovery capacity and pain sensation^26,27^.

The participants were randomly divided into three experimental groups (Table 1) with different textures and hardness of rollers and one control group (resting passively)—each consisting of 15 individuals:

- STH—rolling with a smooth roller—density—soft;
- G—rolling with a grooved roller—density—mid;
- TP—rolling with a trigger (point) roller—density—hard;
- Pass—passive resting group.

**Table 1:** Biometric characteristics of the participants, taking into account the division into groups (mean ± SD).

	STH n = 15	G n = 15	TP n = 15	Pass n = 15
Age (years)	22.98 ± 2.37	23.69 ± 4.94	22.40 ± 1.76	22.73 ± 1.86
Body mass (kg)	77.37 ± 7.84	79.96 ± 10.69	81.42 ± 6.68	82.47 ± 11.40
Body height (cm)	183.03 ± 5.88	179.55 ± 10.44	181.39 ± 6.32	180.66 ± 7.20

### Procedures

In the study, the selected independent variables were the texture and hardness of the applied roller during rolling of specific muscle groups in the lower limbs. The assessment of regeneration effectiveness was conducted using biochemical measurements. For the evaluation of the immediate impact of rolling on post-exertion recovery, measurements of lactate concentration in the blood were taken using the LactateScout + analyzer (ensLab GmbH, Leipzig, Germany)^28^. Furthermore, participants in the experiment assessed the level of subjectively perceived pain in muscles (calves, quadriceps, hamstrings, and glutes) at 24, 48, and 72 h after the completion of the exercise trial. A visual analog scale (VAS) ranging from 0 to 10 was used for measurements, where zero represents no pain, and 10 indicates the most intense, unbearable pain^29^. To avoid relying solely on subjective assessments of SMR effectiveness, creatine kinase levels were measured at 24, 48, and 72 h after the exercise trial by a laboratory technician, using the Dr. Lange LP 420 device (Dr. Lange, Konisburg, Germany) and a liquid two-component reagent for determining CK activity in serum or plasma^30^.

The experiment was conducted at a fixed time between 10 AM and 1 PM to exclude the influence of circadian rhythm on the obtained results^31^. The study began with a 15-min passive adaptation to the environmental conditions in sports sportswear suitable for exercise. After taking measurements according to the adopted protocol (Table 2), a exertion trial was performed, consisting of executing maximal vertical jumps from a full squat position (SJ) for 1 min. Implementing such an effort allowed for inducing significant fatigue, leading to substantial lactic acidosis and the formation of micro-damage to muscle fibers^12^.

**Table 2:** Timeline of study protocol.

Time [min]	Description	
15	Thermal adaptation to external condition	
1	Blood lactate measurements	
1	**Exercise test—vertical jumps from a full squat for 1 min**	
1	Blood lactate measurements	
10	**Foam rolling STH, G, TP**	**Pass**
19	Passive rest	Passive rest
	**Measurements after 30 min from the moment the exercise ends**	
1	Blood lactate measurements	
24 h	CK and VAS measurements	
48 h		
72 h		

During the exertion, participants were motivated to maximize their effort, especially in the last 20 s of work. Immediately after the SJ, a capillary blood sample was taken from the fingertip to determine the blood lactate concentration. Subsequently, participants carried out tasks according to the procedure characteristic of their assigned experimental group. Three groups of 15 individuals each rolled according to the study protocol. Participants were instructed on the technique, order, and rhythm of rolling before the exertion trial. The control group rested passively. Proper execution of rolling was supervised by an experience expert in this area. Self-myofascial release targeted five muscle groups, each rolled for 120 s. Participants began on the shin by rolling posterior side, then moved to the anterior side. Later participants continued by working on relaxing the posterior muscle compartment of the thigh before moving to the anterior compartment. Finally, they massaged the gluteal muscle group. The appropriate width of the roller ensured work on the entire surface of both limbs simultaneously. The rolling technique involved moving the roller from the distal to the proximal attachment and back on both limbs, starting from the farthest muscle groups (each move lasting about 1–1.2 s). The pressure force was determined by the participants themselves, applying enough force to ensure the procedure did not cause pain but only a sense of discomfort. This approach was based on studies made by other authors^18,32^.

After completing the rolling, the participants engaged in passive rest for up to 30 min following the cessation of exercise, before undergoing measurements to determine blood lactate concentration once again. Additionally, subjects provided a blood sample, with a specialist-technician measuring the creatine kinase level. Participants had to undergo these procedures at 24, 48, and 72 h after the completion of the exercise. Due to the significant production of creatine kinase after exercise, participants were advised to avoid any intense physical activity until providing the last blood sample. In addition, participants subjectively recorded the perceived pain in individual muscle groups, following the research protocol (4 muscle groups) at 24, 48, and 72 h after completing the exercise. Ultimately, the front compartment of the shin was excluded from the results analysis due to the absence of pain in these muscles among the study participants.

### Statistical analysis

Statistical analysis of the obtained results was performed using the STATISTICA 13 software (Stat. Soft. USA). The assumption of normal distribution of variables was checked using the Shapiro–Wilk test. Equality of variances across groups was assessed using the Levene's test. The assumption of sphericity of variances was also verified in the repeated measures procedure (Mauchley's test). To determine the significance of differences in the mean values of the examined indicators in successive measurements, a repeated measures analysis of variance (ANOVA) was applied, with the Bonferroni post hoc test. For checking changes between measurements, one-way ANOVA (Bonferroni post hoc test) was used. Effect size measures utilized eta-squared statistics (η^2^): small effect, < 0.10; medium effect, 0.10–0.40; and large effect, > 0.40^33^. A significance level of p < 0.05 was established for all analyses.

A minimum required total sample size of 52 for entire group, was calculated using the G*Power software for ANOVA repeated measures between factors (α = 0.05; number of groups: 4; number of measurements: 3; effect size f = 0.5; actual power = 0.959).

## Results

### Changes of blood lactate level—immediate recovery effect

The analysis of the mean values of blood lactate concentration revealed statistically significant changes over time and among the studied groups. A statistically significant interaction between the groups and time was also observed (Table 3). Analyzing the results of the Bonferroni post-hoc test, no statistically significant differences (p < 0.05) were found between groups in the same measurement for values before exercise, as well as immediately after its completion. The average content of the first measurement ranged from 1.86 mmol/L in the STH group to 2.22 mmol/L in the Pass group. In the "Post" measurement, the blood lactate concentration ranged from 11.08 mmol/L in the STH group to 12.39 mmol/L in the Pass group (Table 3).

**Table 3:** Mean lactate value [mmol/L] in blood, ± SD, (95% CI) and results of ANOVA.

	LA			Factor	F	p	η^2^
	Before	After	30 min				
STH	1.86 ± 0.46 (1.6–2.12)	11.08 ± 1.27 (10.37–11.78)	5.21 ± 1.14 (4.57–5.84)*	Time	1349.007	0.000	0.960
G	2.06 ± 0.32 (1.88–2.24)	11.18 ± 1.43 (10.39–11.98)	5.3 ± 1.82 (4.29–6.31)*	Group	63.494	0.001	0.258
TP	2.15 ± 0.57 (1.83–2.46)	12.2 ± 1.42 (11.42–12.99)	5.69 ± 1.6 (4.81–6.58)*				
Pass	2.22 ± 0.75 (1.81–2.63)	12.39 ± 1.73 (11.43–13.35)	7.65 ± 1.43 (6.86–8.44)	Time*Group	4.556	0.000	0.196

*Significant difference to group pass 30 min after the end of exercise.

**Figure 1:**
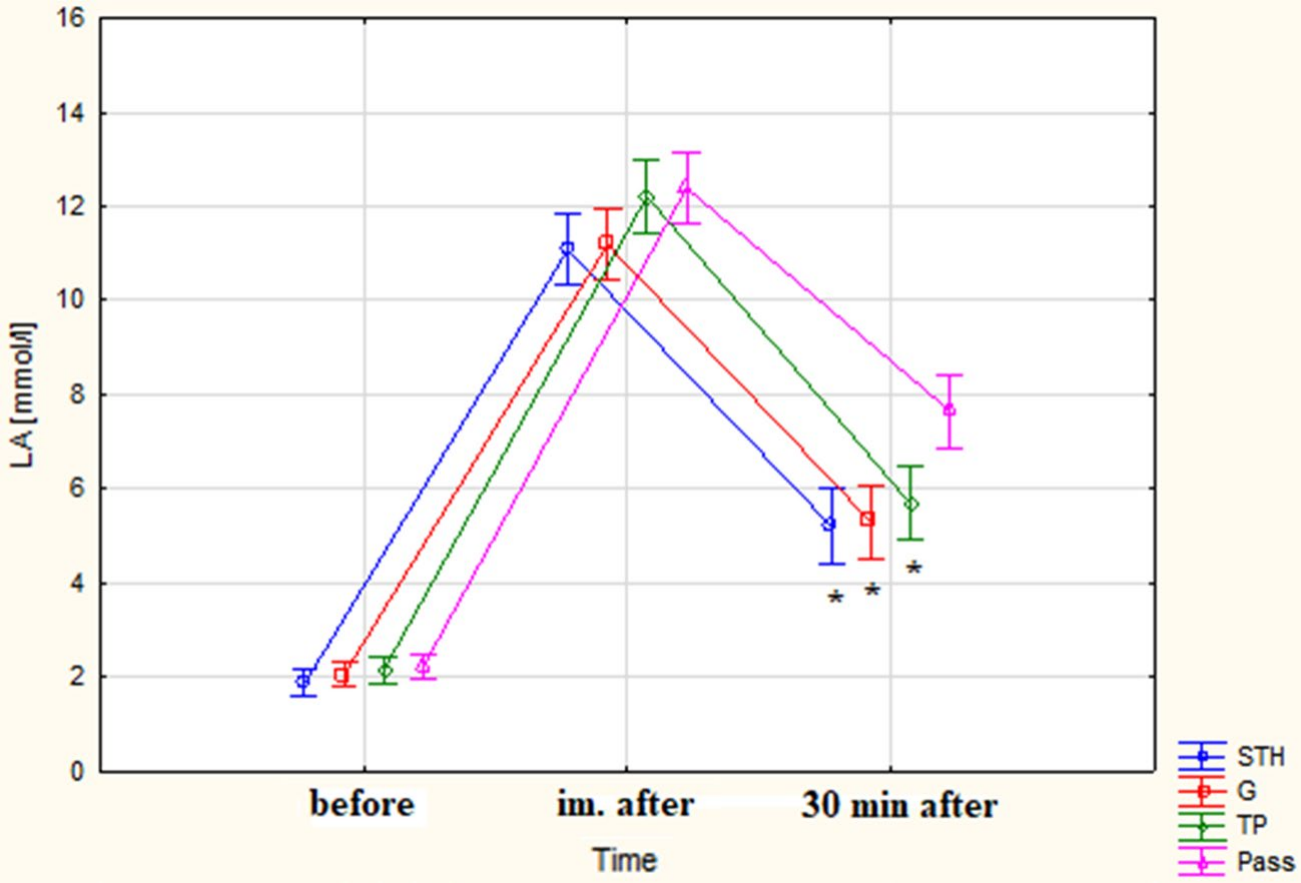
Mean blood lactate concentrations in successive measurements ± SD (*statistical difference from the Pass group, p < 0.05). Current effect: F(6, 112) = 4.5657, p = 0.00035. Vertical bars indicate 0.95 confidence intervals).

The analysis of the average blood lactate level 30 min after exercise revealed a statistically significant difference for all rolling groups compared to the Pass: STH (p < 0.001), G (p < 0.001), TP (p = 0.035). The average values for the rolling groups ranged from 5.21 to 5.69 mmol/L while in the Pass group, a concentration value of 7.65 mmol/L was recorded (Fig. 1).

One-way analysis of variance (ANOVA) did not show significant differences between groups in the "After-Before" measurements (Table 4). However, in the "30 min—Post" measurements, only the result between the TP group and Pass group was significantly different (p = 0.019 in the Bonferroni post-hoc test).

**Table 4:** Mean value of lactate difference [mmol/L] in blood, ± SD, (95% CI) and results of one-way ANOVA.

	Before-after	30 min-after	30 min-before			
STH	9.22 ± 1.35 (8.47–9.97)	− 5.87 ± 1.38 (− 6.63 to − 5.11)	3.35 ± 1.3 (2.63–4.07)*			
G	9.13 ± 1.62 (8.23–10.02)	− 5.88 ± 1.78 (− 6.87 to − 4.9)	3.24 ± 1.82 (2.23–4.25)*			
TP	10.06 ± 1.39 (9.29–10.83)	− 6.51 ± 1.27 (− 7.21 to − 5.81)*	3.55 ± 1.48 (2.73–4.37)*			
Pass	10.17 ± .36 (9.42–10.92)	− 4.74 ± 1.25 (− 5.43 to − 4.04)	5.43 ± 1.21 (4.76–6.1)			

Factor	Free parameter	Group	Free parameter	Group	Free parameter	Group
F	2529.34	2.306	930.294	3.317	383.722	6.113
p	0.000	0.086	0.000	0.026	0.000	0.001
η^2^	0.978	0.109	0.943	0.151	0.873	0.247

*Significant difference from the Pass group.

ANOVA showed significant differences were observed between the groups, post-hoc analysis revealed significant statistical differences in the TP group—3.55 mmol/L (p = 0.037), STH—3.35 mmol/L (p < 0.004), G—3.24 mmol/L (p < 0.002) compared to the Pass group. These differences indicate a significantly faster pace of recovery in the rolling groups compared to the Pass group.

### Creatine kinase changes—prolonged recovery effect

The gathered data did not show statistically significant differences between the results in individual measurements (Table 5). No changes were even noted over time ((F = 1.184; p < 0.311; η^2^ = 0.023). No statistically significant differences were observed between the groups in the mean CK concentration in the blood between measurements.

**Table 5:** Mean creatine kinase—CK [U/L] in blood, ± SD, (95%CI) at 24, 48, and 72 h after exercise, and results of ANOVA analysis.

	CK			Factor	F	P	η^2^
	24 h	48 h	72 h				
STH	112.73 ± 38.84 (90.89–133.91)	112.73 ± 85.36 (65.46–160.00)	123.40 ± 143.39 (43.99–202.81)	Time	1.181	0.311	0.023
G	142.69 ± 77.55 (95.83–189.56)	255.69 ± 295.89 (76.89–434.50)	218.38 ± 265.13 (58.17–378.60)	Group	2.585	0.063	0.132
TP	119.67 ± 52.90 (90.37–148.96)	100.80 ± 50.53 (72.82–128.78)	96.57 ± 97.67 (40.18–152.96)				
Pass	93.79 ± 66.99 (55.11–132.46)	121.07 ± 128.36 (46.96–195.19	116.64 ± 180.30 (12.54–220.75)	Time	1.181	0.311	0.023

### Subjective pain assessment using the VAS scale

**Figure 2:**
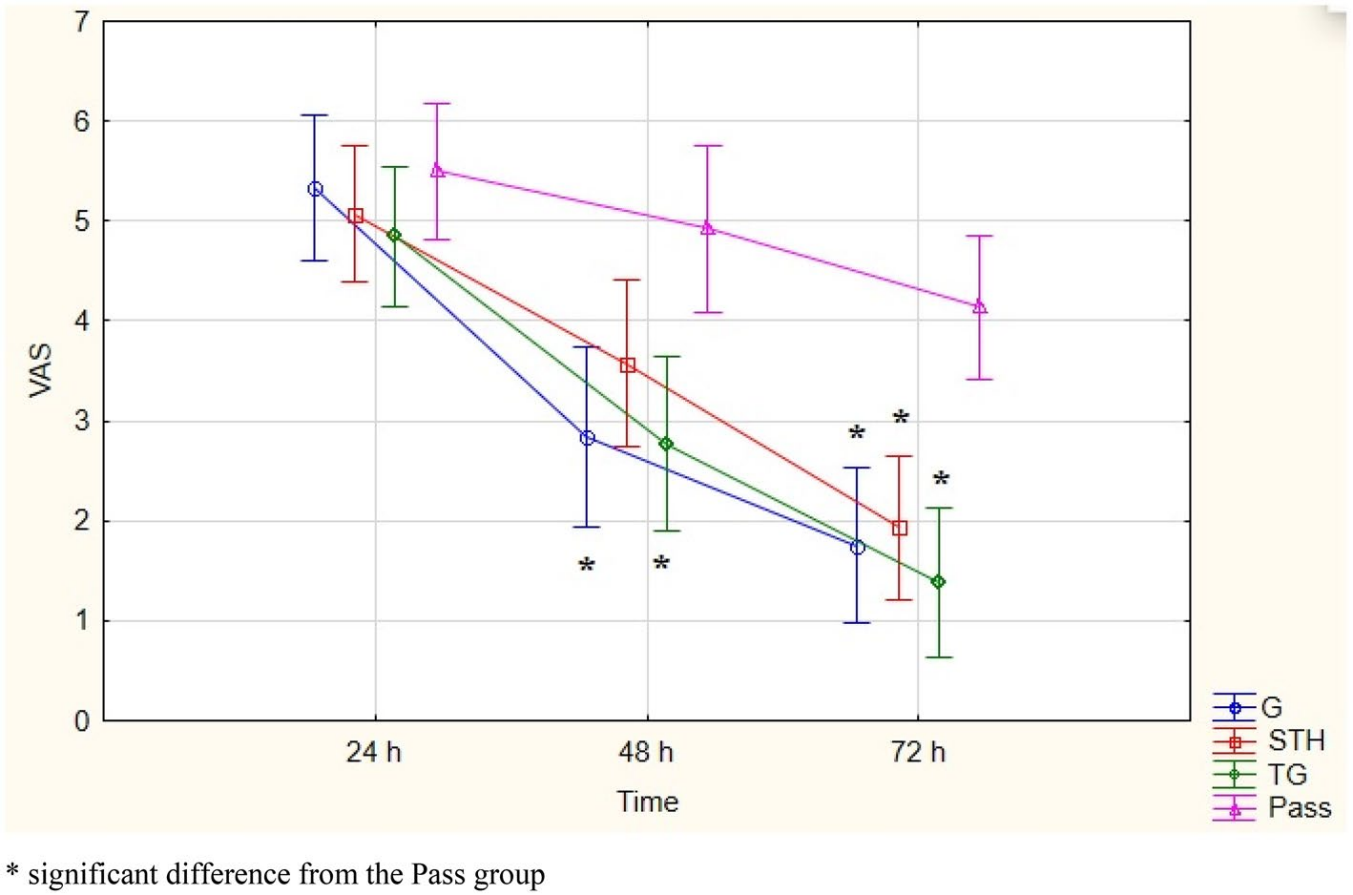
Mean values of VAS for the quadriceps, ± SD, (95% CI) at 24, 48, and 72 h after exercise, and results of ANOVA analysis. Current effect: F(6, 98) = 2.6024: p = 0.02205. Vertical bars indicate 0.95 confidence intervals. *Significant difference from the Pass group.

Statistically significant changes of VAS were of observed over time, as well as among the studied groups (Fig. 2).Analyzing the results of the Bonferroni post-hoc test for the average VAS values in the quadriceps muscles 24 h after the end of exercise, no statistically significant differences were found between groups. Statistically significant values were noted between the G group (p = 0.013) and TP group (p = 0.006) compared to the Pass group at 48 h, and between the STH group (p = 0.003); G group (p = 0.001); TP group (p < 0.001) compared to the Pass group at 72 h. Based on statistical data, a strong effect of time η^2^ = 0.578 on the VAS variable for the quadriceps muscle was observed, as well as a very clear influence of the group η^2^ = 0.399 and their interaction η^2^ = 0.137 (Table 6).

**Table 6:** Mean values of VAS changes for the quadriceps, ± SD, 95% CI, and results of one-way ANOVA analysis.

	ΔVAS quadriceps					
	48–24 h	72–48 h	72–24 h			
STH	− 1.5 ± 1.99 (− 2.64/− 0.35)	− 1.64 ± 0.84 (− 2.12/− 1.15)	− 3.14 ± 1.83 (− 4.2/− 2.08)			
G	− 2.5 ± 2.02 (− 3.78/− 1.21)	− 1.08 ± 0.79 (− 1.58/− 0.57)	− 3.58 ± 1.62* (− 4.61/− 2.55)			
TP	− 2.07 ± 1.55 (− 3.01/− 1.13)	− 1.38 ± 1.26 (− 2.14/− 0.62)	− 3.46 ± 1.85* (− 4.58/− 2.34)			
Pass	− 0.57 ± 2.53 (− 2.03/0.89)	− 0.78 ± 2.25 (− 2.09/0.51)	− 1.35 ± 2.13 (− 2.58/− 0.12)			

Factor	Free parameter	Group	Free parameter	Group	Free parameter	Group
F	34.212	2.159	38.161	0.920	124.489	4.126
P	0.000	0.105	0.000	0.438	0.000	0.011
η^2^	0.411	0.117	0.438	0.053	0.718	0.202

*Significant difference from the Pass group.

Significant differences between groups were observed in the 72–24 h measurement. Post-hoc test results showed a difference between the G group (p = 0.025) and TP group (p = 0.033) compared to the Pass group.

## Discussion

The aim of the study was to examine the influence of rollers with different textures and densities on the speed of blood lactate removal after intense exercise and to determine the effect of a single SMR intervention on the speed of recovery at 24, 48, and 72 h, using the assessment of CK concentration and subjective VAS evaluation.

### Immediate recovery (LA)

In the conducted study, the analysis of the obtained maximal post-exercise LA values showed that they were similar to the results obtained by other researchers^12,34,35^ approximately around 11 ± 2 mmol/L. The comparison of average LA values at 30 min did not reveal statistically significant differences between the rolling groups despite the use of rollers with different surface textures and foam densities.

In this study, the analysis also included the differences in the average decrease in LA between the second and third measurements. The calculations did not reveal significant differences between the rolling groups and the Passive Rest (Pass) group. However, the average values of lactate decrease in all rolling groups were higher than in the Pass group, indicating an accelerated lactate utilization. There was also a statistically significant difference between the third and first measurements in the rolling groups compared to the values of the Pass group. The smaller difference between the first and last measurements in these groups indicates a much more efficient return of this indicator to the initial values, i.e., before exercise. Faster lactate clearance is associated with increased blood and lymph flow induced by the mechanical pressure of the roller on tissue structures^32^. The results obtained suggest the superiority of an active form supporting immediate recovery using a roller over passive rest, confirming the findings of other authors^34^. Moreover, literature provides many examples confirming the advantage of other forms of active recovery over passive rest (e.g.^36–38^). Due to movement performed during rolling by the muscles themselves can be treated as an active form of recovery.

However, a difference was demonstrated between all rolling groups and the passive rest group. Similar phenomena were observed by Adamczyk et al.^12^, who applied STH and G rollers in their study, comparing the effects with a passively resting group. This phenomenon can be explained by the increased blood flow in blood vessels during rolling, leading to accelerated removal of LA from the blood^10^. Therefore, the type of roller surface may not be significant, but rather the area subjected to the procedure and the starting point, in line with the anatomical structure of veins and the direction of blood flow. Additionally, some researchers indicate the applied pressure as a significant factor resulting from the intervention, as well as the frequency of application^39^. The recommended frequency is around 50 strokes per minute, or approximately 1.2 s for a full cycle from distal to proximal and back. The recommended pressure force is related to the participant's body weight, applied in a way to induce discomfort rather than pain^40^. Unfortunately, to the best of our knowledge, there is no more scientific studies that simultaneously compared the impact of different rollers on post-exercise LA levels. However, there are publications where a single type of roller was used, and its impact on lactate levels was measured. One such report is the study by Kalén et al.^34^, examining LA before, immediately after, and 25 min after exercise. The authors compared the regeneration rate in the rolling group, active resting (running), and passive rest group. In the study, a roller with high density was used, but unfortunately, the texture of this tool was not mentioned. In the conducted experiment, active regeneration and rolling led to a significantly faster decrease in LA levels compared to the passive rest group. No statistically significant differences were found between the rolling and actively resting groups. Similarly, Alonso-Calvete et al.^35^ obtained significantly lower average LA values between 9 min after exercise and immediately post-exercise, between the rolling groups and the passively resting group. In their study, they used Hard-density rollers and STH-type texture, but the first group performed SMR with the vibration function activated, while the second group did not. No significant changes between the rolling groups were found, but the effects compared to the passively resting group were greater for the rolling group. Kappenstein et al.^36^ in their study on post-sprint exercise lactate levels, lasting 5 s, repeated 5 times in two series, obtained a statistically significant difference in LA decrease between the passively resting group and the active group at 15 min.

On the other hand, available literature also includes studies where scientists argue that there are no significant differences in LA decrease between groups actively and passively resting^41^. The study by Losnegard et al.^42^ conducted on a group of elite cross-country skiers did not indicate significantly lower LA values in the actively resting group compared to athletes resting passively. Contradictory results may arise from the diversity of applied physical efforts, tools, and forms of recovery, as well as the time allocated for rest. The analyzed literature predominantly shows a more positive impact of rolling on processes related to the pace of recovery after short-term intense physical efforts which can be explained by the physiological mechanism of fluid movement, resulting from the pressure caused by the roller. Additionally, roller massage can reduce creatine kinase, which helps in reducing pain or DOMS^43^.

### Prolonged recovery (CK and VAS)

The gathered material showed no significant differences between measurements and groups. This suggests a minimal influence of a single rolling intervention on the CK indicator or a lack of connections between rolling and CK levels. It also seems that measuring at the so-called zero point (rest) would be useful for future studies. Moreover, the absence of significant decreases between values on consecutive days indicates the need to extend the research with additional measurements at 96 or even 120 h^44^. In the presented results, the highest VAS values were obtained at 24 or 48 h in all studied groups. These values are reflected in many studies evaluating the effectiveness of SMR on Delayed Onset Muscle Soreness (DOMS)^8,12,15,45,46^. A thorough assessment of VAS and obtaining statistically significant differences between all rolling groups and the Passive Rest (Pass) group on the third day, as well as between the G and TP groups and the Pass group on the second day within the quadriceps muscles, allows the conclusion that rolling can bring a beneficial effect after maximal anaerobic effort. Within the uniform duration of the procedure in the conducted research, no differences were found between the rolling groups, which justifies the statement that the type of roller surface does not matter. This reinforces a similar conclusion reached by Adamczyk et al.^12^.

The collected data align with the results of other researchers who found the highest CK values after intense short-term anaerobic exercise at 24 or 48 h^47,48^ however the availability of studies evaluating the impact of rolling on CK levels after exercise is quite limited. Visconti et al.^49^ following the protocol of MacDonald et al.^8^, obtained statistically significantly higher CK values between the measurement at 48 h vs. before exercise and at 48 h vs. 24 h in both studied groups (rolling and passive rest). However, no differences between groups were found, which may confirm the lack of a connection between SMR and post-exercise CK concentration with a high load however the protocol used a high-density foam roller, but the texture was not specified. Kuswahyudi et al.^45^ compared CK levels at 24 h between a group rolling with a Grid-type tool (no information on density), immersing in cold water, and jogging at a slow pace. Researchers found no differences between these three forms of active recovery in CK values on the first day after exercise. The obtained values put all three forms of recovery assistance on an equal footing.

Yanaoka et al.'s study^50^ found no differences in the effects produced by foam rollers of MED and Hard densities. A two-minute intervention with different rollers did not result in differences in muscle soreness (on a scale of 0–10) and serum CK concentration. These results demonstrated the positive impact of rollers on the speed of recovery processes, and different densities lead to similar effects. Cheathman and Stull^51^ investigated the influence of rollers with the same density but different surfaces (Smooth, Grid, and Multilevel) on passive knee range of motion and pain perception in the quadriceps muscle (PPT). In this case, statistically significant changes were found between measurements before and after in all types of rollers, but without significant differences between the rollers. The more pronounced action of Grid and Multilevel (TG in our case) rollers may result from the surface structure, which causes greater tissue deformation. These changes are induced by both mechanical and neurophysiological effects. However, 72 h after exercise, all types of rollers showed significantly lower differences in the VAS scale compared to the Pass group, without differences between the rollers.

Analyzing the results of studies evaluating the impact of rolling on Delayed Onset Muscle Soreness (DOMS), MacDonald et al.^8^ obtained significantly lower NRS scale results between the rolling group and the control group at 24, 48, and 72 h after the exercise. Mustafa et al.^52^, using the same study protocol as MacDonald et al.^8^, also achieved statistically better results in subsequent days between the rolling group and the group resting passively. The presented studies provide evidence of the beneficial impact of rolling in counteracting DOMS which can be explained by the mechanical pressure which roller exerts on muscles previously loaded by exercise. It can help increase blood flow by raising arterial pressure and also increase muscle temperature as a result of conduction and friction^53^. Additionally, the mechanical pressure on the muscle is expected to alter nerve excitability, measured by the Hoffmann reflex (neurological mechanism). The quality of recovery is undoubtedly influenced by changes in parasympathetic activity and hormone levels (e.g., measured by cortisol levels), causing a relaxation response (physiological mechanisms)^54^, which can also be achieved by using the SMR technique^55^.

### Time of treatments

SMR procedures are most often performed in time intervals of 30, 60, and 120 s. Undoubtedly, this is based on manual therapy protocols. There is a lack of studies in the literature that clearly indicate significantly better benefits from using any of these three treatment durations. However, Patel et al.^56^ noted that most evaluations of the impact of SMR treatments used intervention durations between 60 and 120 s. Additionally, researchers studying muscle tissue and fascia suggest that only interventions lasting 90–120 s induce adaptive responses to the pressure applied during SMR^57^. Furthermore, Couture et al.^39^ stated that rolling for less than 2 min is insufficient to achieve an improvement in the range of motion in the knee joint. Moreover, some researchers applied rolling treatments in series, summing to a total time of 120 s^8,58^. Monteiro et al.^5^ concluded that better results were observed in each group using foam rollers compared to equivalent groups using manual rollers, but only the group with a 120-s treatment showed a statistically significant difference compared to its 60-s manual roller.

## Summary and conclusions

Our results confirm the effectiveness of foam rolling in supporting both immediate and prolonged recovery. This is exceptionally important from an athlete's perspective and their readiness between subsequent training sessions. The obtained data, as well as the results of other researchers, indicate a lack of significance regarding the type of texture and density of the applied roller, especially when it comes to GRID and MULTILEVEL rollers with MED or HARD density. The conducted studies point to a significantly better post-exertional recovery pace after using a foam rolling treatment lasting at least 120 s. With such treatment duration, the texture and hardness of the applied tool had no significance. When assessing post-exertional fatigue, it is also important to examine other biochemical indicators that will determine, in a broader and more accurate scope, the impact of training loads on the internal strain of the body, such as serotonin, cortisol, epinephrine, prolactin, testosterone, luteinizing hormone, and interleukin-6^59^.

## Data Availability

The datasets (generated during and/or analysed during the current study) are gathered under this link Database.xlsx and are available from the corresponding author on reasonable request.
